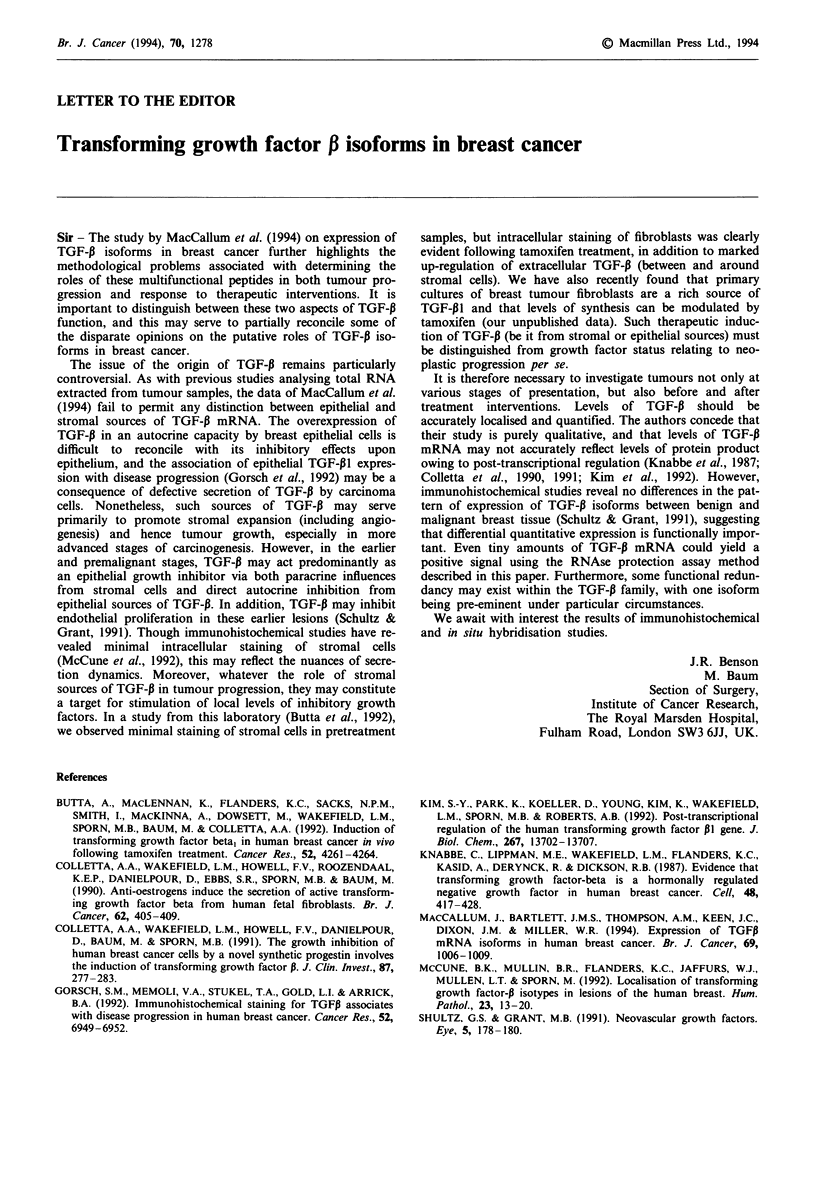# Transforming growth factor beta isoforms in breast cancer.

**DOI:** 10.1038/bjc.1994.489

**Published:** 1994-12

**Authors:** J. R. Benson, M. Baum


 © Macmillan Press Ltd., 1994

### LETTER TO THE EDITOR

### Transforming growth factor β isoforms in breast cancer

**Sir** – The study by MacCallum *et al.* (1994) on expression of TGF-β isoforms in breast cancer further highlights the methodological problems associated with determining the roles of these multifunctional peptides in both tumour progression and response to therapeutic interventions. It is important to distinguish between these two aspects of TGF-β function, and this may serve to partially reconcile some of the disparate opinions on the putative roles of TGF-β isoforms in breast cancer.

The issue of the origin of TGF-β remains particularly controversial. As with previous studies analysing total RNA extracted from tumour samples, the data of MacCallum *et al.* (1994) fail to permit any distinction between epithelial and stromal sources of TGF-β mRNA. The overexpression of TGF-β in an autocrine capacity by breast epithelial cells is difficult to reconcile with its inhibitory effects upon epithelium, and the association of epithelial TGF-β1 expression with disease progression (Gorsch *et al.*, 1992) may be a consequence of defective secretion of TGF-β by carcinoma cells. Nonetheless, such sources of TGF-β may serve primarily to promote stromal expansion (including angiogenesis) and hence tumour growth, especially in more advanced stages of carcinogenesis. However, in the earlier and premalignant stages, TGF-β may act predominantly as an epithelial growth inhibitor via both paracrine influences from stromal cells and direct autocrine inhibition from epithelial sources of TGF-β. In addition, TGF-β may inhibit endothelial proliferation in these earlier lesions (Schultz & Grant, 1991). Though immunohistochemical studies have revealed minimal intracellular staining of stromal cells (McCune *et al.*, 1992), this may reflect the nuances of secretion dynamics. Moreover, whatever the role of stromal sources of TGF-β in tumour progression, they may constitute a target for stimulation of local levels of inhibitory growth factors. In a study from this laboratory (Butta *et al.*, 1992), we observed minimal staining of stromal cells in pretreatment samples, but intracellular staining of fibroblasts was clearly evident following tamoxifen treatment, in addition to marked up-regulation of extracellular TGF-β (between and around stromal cells). We have also recently found that primary cultures of breast tumour fibroblasts are a rich source of TGF-β1 and that levels of synthesis can be modulated by tamoxifen (our unpublished data). Such therapeutic induction of TGF-β (be it from stromal or epithelial sources) must be distinguished from growth factor status relating to neoplastic progression *per se*.

It is therefore necessary to investigate tumours not only at various stages of presentation, but also before and after treatment interventions. Levels of TGF-β should be accurately localised and quantified. The authors concede that their study is purely qualitative, and that levels of TGF-β mRNA may not accurately reflect levels of protein product owing to post-transcriptional regulation (Knabbe *et al.*, 1987; Colletta *et al.*, 1990, 1991; Kim *et al.*, 1992). However, immunohistochemical studies reveal no differences in the pattern of expression of TGF-β isoforms between benign and malignant breast tissue (Schultz & Grant, 1991), suggesting that differential quantitative expression is functionally important. Even tiny amounts of TGF-β mRNA could yield a positive signal using the RNAse protection assay method described in this paper. Furthermore, some functional redundancy may exist within the TGF-β family, with one isoform being pre-eminent under particular circumstances.

We await with interest the results of immunohistochemical and *in situ* hybridisation studies.

J.R. Benson
M. Baum
Section of Surgery,
Institute of Cancer Research,
The Royal Marsden Hospital,
Fulham Road, London SW3 6JJ, UK.

### References

BUTTA, A., MACLENNAN, K., FLANDERS, K.C., SACKS, N.P.M., SMITH, I., MACKINNA, A., DOWSETT, M., WAKEFIELD, L.M., SPORN, M.B., BAUM, M. & COLLETTA, A.A. (1992). Induction of transforming growth factor $beta_1$ in human breast cancer *in vivo* following tamoxifen treatment. *Cancer Res.*, **52**, 4261–4264.

COLLETTA, A.A., WAKEFIELD, L.M., HOWELL, F.V., ROOZENDAAL, K.E.P., DANIELPOUR, D., EBBS, S.R., SPORN, M.B. & BAUM, M. (1990). Anti-oestrogens induce the secretion of active transforming growth factor beta from human fetal fibroblasts. *Br. J. Cancer*, **62**, 405–409.

COLLETTA, A.A., WAKEFIELD, L.M., HOWELL, F.V., DANIELPOUR, D., BAUM, M. & SPORN, M.B. (1991). The growth inhibition of human breast cancer cells by a novel synthetic progestin involves the induction of transforming growth factor β. *J. Clin. Invest.*, **87**, 277–283.

GORSCH, S.M., MEMOLI, V.A., STUKEL, T.A., GOLD, L.I. & ARRICK, B.A. (1992). Immunohistochemical staining for TGFβ associates with disease progression in human breast cancer. *Cancer Res.*, **52**, 6949–6952.

KIM, S.-Y., PARK, K., KOELLER, D., YOUNG, KIM, K., WAKEFIELD, L.M., SPORN, M.B. & ROBERTS, A.B. (1992). Post-transcriptional regulation of the human transforming growth factor β1 gene. *J. Biol. Chem.*, **267**, 13702–13707.

KNABBE, C., LIPPMAN, M.E., WAKEFIELD, L.M., FLANDERS, K.C., KASID, A., DERYNCK, R. & DICKSON, R.B. (1987). Evidence that transforming growth factor-beta is a hormonally regulated negative growth factor in human breast cancer. *Cell*, **48**, 417–428.

MACCALLUM, J., BARTLETT, J.M.S., THOMPSON, A.M., KEEN, J.C., DIXON, J.M. & MILLER, W.R. (1994). Expression of TGFβ mRNA isoforms in human breast cancer. *Br. J. Cancer*, **69**, 1006–1009.

MCCUNE, B.K., MULLIN, B.R., FLANDERS, K.C., JAFFURS, W.J., MULLEN, L.T. & SPORN, M. (1992). Localisation of transforming growth factor-β isotypes in lesions of the human breast. *Hum. Pathol.*, **23**, 13–20.

SHULTZ, G.S. & GRANT, M.B. (1991). Neovascular growth factors. *Eye*, **5**, 178–180.