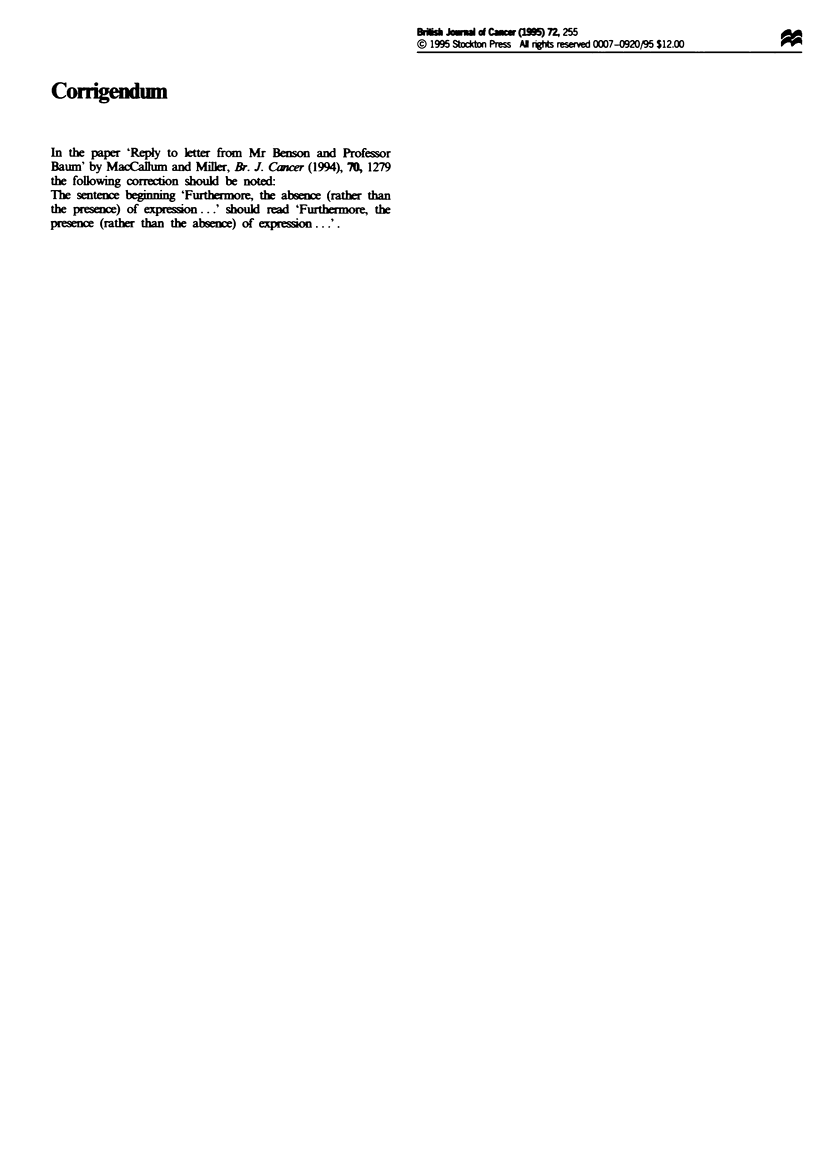# Corrigendum

**Published:** 1995-07

**Authors:** 


© 1995 Stockton Press All rights reserved 0007-0920/95 $12.00

### Corrigendum

In the paper 'Reply to letter from Mr Benson and Professor Baum' by MacCallum and Miller, *Br. J. Cancer* (1994), **70**, 1279 the following correction should be noted:

The sentence beginning 'Furthermore, the absence (rather than the presence) of expression . . .' should read 'Furthermore, the presence (rather than the absence) of expression . . .'.